# Causal associations between osteoporosis and HBV infection across Asian and European populations: evidence from Mendelian randomization and colocalization analysis

**DOI:** 10.3389/fendo.2024.1419303

**Published:** 2025-01-22

**Authors:** Zhengnan Li, Jiarui Cao, Ke Li, Yixin Wu, Zhanpeng Luo, Rui Cao, Zhiheng Cheng, Zhendong Tian, Yiyang Han, Yuping Lai, Bangqi Wang, Shen Chen

**Affiliations:** ^1^ Department of Sports Medicine, Ganzhou People’s Hospital, Ganzhou, Jiangxi, China; ^2^ Queen Mary School, Medical College, Nanchang University, Nanchang, China; ^3^ The Huankui Academy, Nanchang University, Nanchang, Jiangxi, China; ^4^ State Key Laboratory of Molecular Oncology and Department of Radiation Oncology, National Cancer Center/National Clinical Research Center for Cancer/Cancer Hospital, Chinese Academy of Medical Sciences and Peking Union Medical College, Beijing, China; ^5^ The Key Laboratory of Geriatrics, Beijing Institute of Geriatrics, Institute of Geriatric Medicine, Chinese Academy of Medical Sciences, Beijing Hospital/National Center of Gerontology of National Health Commission, Beijing, China

**Keywords:** osteoporosis, HBV, STAT4, Mendelian randomization, Colocalization analysis

## Abstract

**Introduction:**

Clinical studies have demonstrated a potential association between chronic hepatitis caused by hepatitis B virus (HBV) infection and osteoporosis. However, the causal relationship between HBV infection and osteoporosis remains to be determined.

**Methods:**

We investigated whether HBV infection is causally associated with osteoporosis using Mendelian randomization (MR) in East Asian and European populations, respectively. The data we utilized were obtained from the genome-wide association studies (GWAS) database. Various MR methods, including inverse variance weighted (IVW), MR Egger, weighted median, simple median and simple mode were employed to estimate the association between HBV infection and osteoporosis. Heterogeneity analysis and sensitivity tests were performed to ensure the robustness of the results. Bayesian co-localization (coloc) analysis was also applied to calculate the posterior probability of causal variants and to identify common genetic variants between HBV infection and osteoporosis.

**Results:**

MR analysis indicated that HBV infection increased the risk of osteoporosis onset in two East Asian cohort (IVW, OR = 1.058, 95% CI = 1.021 to 1.097, P = 0.002 and OR = 1.067, 95% CI = 1.029 to 1.106, P < 0.001). However, a clear effect of genetic susceptibility to HBV on the enhanced risk of osteoporosis was not observed in two European cohort (IVW, OR = 1.000, 95% CI = 0.999 to 1.001, P = 0.171 and OR = 1.003, 95% CI = 0.981 to 1.025, P = 0.780). Additional MR methods and sensitivity analyses further validated the reliability and robustness of our results. Bayesian co-localization analysis revealed co-localization of HBV infection and osteoporosis on STAT4 at rs11889341based on East Asian GWAS data.

**Conclusions:**

Our study identified a causal relationship between HBV infection and osteoporosis in East Asian and European populations. These results provided strong evidence that HBV infection augmented the risk of developing osteoporosis in East Asian populations and provided novel therapeutic targets.

## Introduction

Osteoporosis is the most prevalent bone metabolic disease characterized by decreased bone mineral density (BMD), structural deterioration of bone tissue as well as increased risk of fracture (1). It is estimated that osteoporosis affects more than 200 million people worldwide and occurs primarily in the elderly, especially in menopausal women (2). In addition, the results of recent research showed that among the subjects they investigated, 95% of patients with osteoporosis had at least one coexisting condition, and they were also at higher risk of developing joint disease, arthritis, chronic low back pain, depression and chronic heart failure (3). Notably, fragility fractures are a major complication of osteoporosis, with a mortality rate of up to 30% within 1 year of fragility fracture occurrence (4). With the tendency of an aging population, osteoporosis will cause a wider impact and more alarming healthcare costs, which will dramatically affect global health and economic issues. Therefore, we are supposed to emphasize the search for potential risk factors of osteoporosis and the exploration of new therapeutic targets.

Hepatitis B virus (HBV) infection is globally widespread and more than 2 billion people are influenced by HBV infection (5). Owing to the hepatophilic nature of HBV, HBV infection may lead to a broad spectrum of liver diseases, including hepatitis, cirrhosis, hepatocellular carcinoma and so on (6). Non-hepatic complications in patients with chronic liver diseases, particularly cirrhosis, include hepatic osteodystrophy. Notably, osteoporosis is the most common form of hepatic osteodystrophy (7). Recent works have explored the potential association between liver diseases arising from chronic hepatitis B and bone health indicators such as BMD and the incidence of osteoporosis. Zhang et al. (8) found that lumbar spine and hip BMD were significantly lower and the occurrence of osteoporosis was much higher in the cirrhotic group of hepatitis B as compared to the control group. Not only that, Schiefke et al. (9) also identified a meaningful reduction in BMD in non-cirrhotic patients with chronic hepatitis B infection. However, Mora et al. (10) measured BMD in untreated adolescents vertically infected with HBV and discovered that neither lumbar spine BMD nor whole-body BMD in HBV-infected patients differed significantly from that of controls. These results were not entirely consistent. In addition, most of the previous studies were observational studies focusing on the correlation between chronic liver disease, especially cirrhosis, and osteoporosis, and mainly used BMD as an observational index. Given the confounding factors and other reasons, they may not be robust enough to establish causality. Therefore, we need an approach to determine whether HBV infection is directly linked to an increased risk of osteoporosis and to explore the biomolecules that are critical in this process.

Currently, Mendelian randomization (MR) is widely recognized as a promising tool for addressing questions in epidemiology and human biology, based on the wide availability of genetic data and the tremendous growth of genome-wide association studies (GWAS) (11). MR employs genetic variants as instrumental variables (IVs) to investigate causal relationships between exposures and outcomes (12). Compared with traditional observational studies, MR is less susceptible to confounding factors and also minimizes the potential risk of reverse causality bias. Thus, MR may present stronger evidence for reflecting causal effects. In this study, we conducted MR analysis with available publicly GWAS datasets of HBV infection and osteoporosis in two populations including East Asia and Europe to explore the causal relationship between HBV infection and osteoporosis. In addition, to verify the MR results and to detect pivotal molecules linking the connection between HBV infection and osteoporosis, we performed Bayesian co-localization (coloc) analysis to calculate the posterior probability of causal variants and to identify shared genes for HBV infection and osteoporosis.

## Methods

### Study design

**Figure 1:**
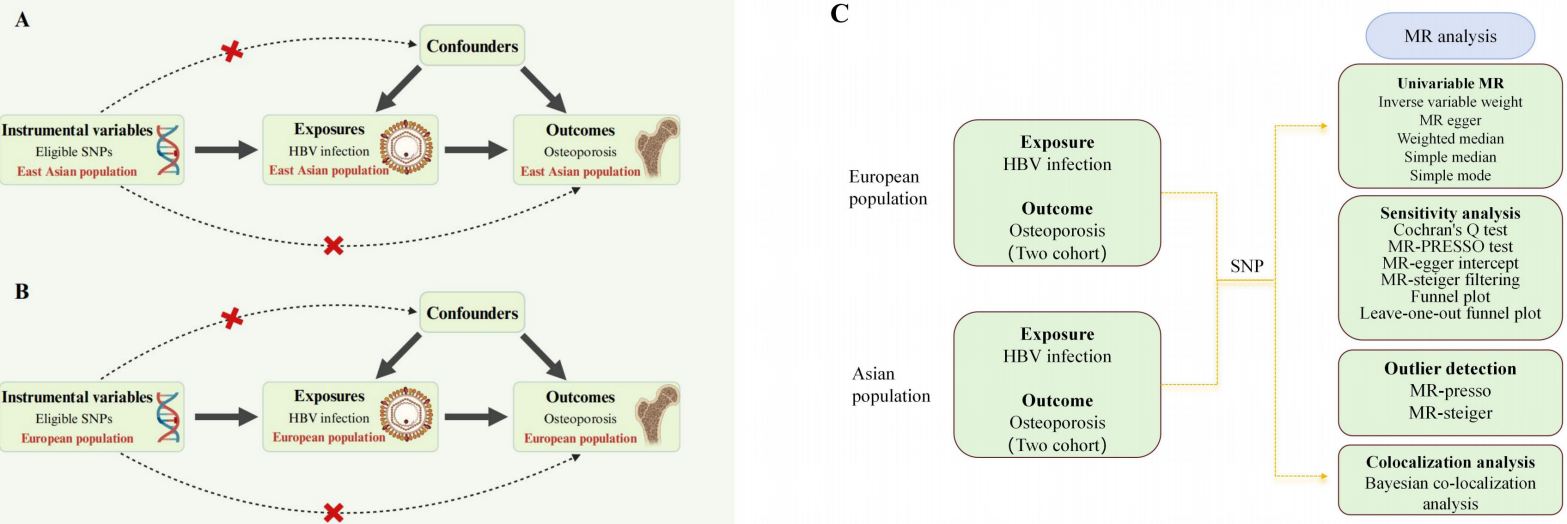
Overall study design based on Mendelian randomization analysis. SNPs, single nucleotide polymorphisms; **(A)** presents the three main Mendelian randomization hypotheses for the Asian population in this study, while **(B)** displays the corresponding hypotheses for the European population. In both figures, the selected instrumental variables are assumed to influence the outcome solely through the exposure and not through any direct association with confounding factors or the outcome itself. **(C)** illustrates the overall workflow of the study. First, we obtained GWAS data and applied five different Mendelian randomization methods to perform two-sample Mendelian randomization in the two populations. This was followed by sensitivity and heterogeneity analyses to assess the stability of the study conclusions, and finally, a colocalization analysis was conducted. HBV, hepatitis B virus.

The study design is described in **Figure 1**. We conducted MR in each of the East Asian and European populations to explore the possible causal relationship between HBV infection and osteoporosis. Based on previous reports in the literature and the GWAS database, we screened single nucleotide polymorphisms (SNPs) closely associated with HBV infection as IVs, including data from both East Asian and European populations. Notably, valid IVs in MR studies are required to fulfill three core assumptions: 1) the association assumption, where IVs need to be directly related to exposure factors, 2) the independence assumption, in which IVs are not related to confounders of exposure and outcome, and 3) the exclusivity assumption, where IVs should only be able to influence outcomes through exposure factors. The GWAS summary dataset cited in this study was approved by the review committee, as provided in the original article. Therefore, this study did not require relevant ethical permission.

This Mendelian randomization study strictly follows the guidelines outlined in the Strengthening the Reporting of Observational Studies in Epidemiology using Mendelian Randomization (STROBE-MR) statement for reporting (**Supplementary Table S5**) (13, 14).

### GWAS data sources and genetic instrument variables

All summary statistics used in this study were obtained from the IEU open gwas project, Finn R11 database and GWAS catalog database (**Table 1**). The GWAS data for Chronic hepatitis B in East Asian populations were mainly derived from the Japan BioBank project, including 8,885,805 SNPs and 212,453 East Asian-origin individuals, among which 1,394 were cases and 211,059 were controls (Chronic hepatitis B dataset: bbj-a-99). Exposure summary data for the European population were based on the study by Sakaue et al. (15), containing 19,079,722 SNPs, and 351,885 European individuals, among which 145 were cases and 351,740 were controls (Chronic hepatitis B infection: ebi-a-GCST90018804). Furthermore, to minimize potential bias due to population heterogeneity, the population characteristics of the MR studies we carried out were corresponding with two different outcome datasets in both populations. Specifically, the pooled data for osteoporosis were also taken from East Asian (Osteoporosis Dataset: bbj-a-13 and ebi-eas-GCST90018667) and European (Non-cancer illness code, self-reported: osteoporosis Dataset: ukb-b-12141 and finngen_R11_M13_OSTEOPOROSIS) populations.

**Table 1:** The characteristics of GWAS studies on this Mendelian randomization.

IEU-ID	Phenotype	SNPs	Cases/controls	Ethnicity	Data link
bbj-a-99	Chronic hepatitis B infection	8,885,805	1,394/211,059	East Asian	https://gwas.mrcieu.ac.uk/datasets/bbj-a-99/
ebi-a-GCST90018804	Chronic hepatitis B infection	19,079,722	145/351,740	European	https://gwas.mrcieu.ac.uk/datasets/ebi-a-GCST90018804/
bbj-a-137	Osteoporosis	8,885,805	7,788/204,665	East Asian	https://gwas.mrcieu.ac.uk/datasets/bbj-a-137/
ukb-b-12141	Osteoporosis	9,851,867	7,547/455,386	European	https://gwas.mrcieu.ac.uk/datasets/ukb-b-12141/
ebi-eas-GCST90018667	Osteoporosis	13,309,275	9,794/168,932	East Asian	https://www.ebi.ac.uk/gwas/studies/GCST90018667
finngen_R11_M13_OSTEOPOROSIS	Osteoporosis	20,093,941	9,046/429,826	European	https://r11.risteys.finregistry.fi/endpoints/M13_OSTEOPOROSIS

SNPs, single nucleotide polymorphisms.

### Instrumental variables

We eliminated IVs that exhibited a substantial correlation with exposure based on the following standards. First, SNPs were associated with exposure at a genome-wide significant level (P < 5 × 10 ^-6^). Moreover, applying the Thousand Genomes Project’s East Asian and European reference panels, we executed an SNP aggregation procedure using the PLINK algorithm, which retained SNPs with linkage disequilibrium (LD) r^2^ < 0.001 in a 10,000-kb window for IVs. Finally, the potency of IVs was evaluated employing the F-statistic: F= (N-k- 1)/k × R^2^/(1 -R^2^), where N denotes the total number of samples in the GWAS study, k is the number of IVs utilized in the analysis, and R2 is used to measure regression model fit. It is worth noting that F<10 is usually considered a weak IV and needs to be removed from the research to prevent weak instrumental bias (16).

### Bayesian co-localization analysis

Bayesian coloc analysis is often utilized to identify whether two phenotypes are motivated by the same causal variant in a region (17). We integrated aggregated data from both exposure and outcome GWAS datasets, then used the “coloc” package with default parameters (https://github.com/chr1swallace/coloc) to calculate the posterior probability of HBV infection and osteoporosis, which is crucial to determine whether they share causal variables. In this study, we focused on the posterior probability of hypothesis 4 (PPH4) in the Bayesian co-localization analysis, which is the posterior probability that HBV infection and osteoporosis are significantly associated with a SNP locus in a certain genomic region and are driven by the same causal variant locus. Typically, PPH4 ≥ 75% is considered evidence of co-localization, suggesting that genetic variants are shared between HBV and osteoporosis and may be involved in regulating their association (18).

### Statistical analysis

In our study, we employed five different approaches to estimate the causal relationship between exposure (hepatitis B) and outcome (osteoporosis). Among them, standard inverse variance weighting (IVW) served as the predominant method of assessment. IVW is based on valid and mutually independent IVs that weight random variable measures by the inverse of the outcome’s variance (se^2^) (19). Additionally, IVW is characterized by regressions that do not account for the presence of an intercept term. Although IVW is regarded as an effective method and is widely used in MR studies, errors may arise when heterogeneity and horizontal pleiotropy are present. Therefore, we performed complementary analyses including MR Egger, weighted median (WM), simple median and simple mode in order to obtain more accurate results. MR-Egger regression takes the presence of an intercept term into account and is capable of assessing horizontal pleiotropy of genetic variation, where a significantly non-zero intercept indicates that the IVs we used may have horizontal pleiotropy (20). WM is the median of the distribution function obtained by ranking all SNP effect values according to their weights, and it yields robust estimates when at least 50% of the information comes from valid IVs (21). The results assessed through the above techniques were deemed statistically significant when the p-value was <0.05. Besides, the effect of the results was estimated by the odds ratio (OR) and the corresponding 95% confidence intervals (CI).

To ensure that the results were stable and reliable, we also tested for heterogeneity, horizontal pleiotropy and sensitivity. We used IVW and MR-Egger to detect heterogeneity between each SNP. Heterogeneity was quantified by calculating the Cochran Q-statistic, and a P-value > 0.05 indicated the absence of heterogeneity and more reliable results (22). Additionally, we visualized outliers using scatterplots and funnel plots. To test for horizontal pleiotropy, we resorted to intercept p-values gained from MR Egger regression as well as MR-Presso global test, and reassessed the causal effect estimates after removing outlier SNPs (23, 24). Moreover, we also conducted leave-one-out analyses to assess the influence of individual SNPs on the causal effects of exposure and outcome. The leave-one-out method indicates reliable results if all error lines are simultaneously on the 0 side after the removal of an SNP (25).

All analyses were performed in the statistical software R (version 4.2.1) utilizing the “TwoSampleMR (version 0.5.7)”, “MRPRESSO (version 1.0)” and “coloc” packages.

## Results

### Instrumental variables for Mendelian randomization

**Supplementary Tables S1**, **S2** provide detailed information on the SNPs used in this study for the East Asian and European populations, respectively. The East Asian population included a total of 27 SNPs, while the European population included 23 SNPs. All of these SNPs had F values greater than 10, indicating the absence of weak instrumentation and effectively demonstrating the potency of the exposure. Moreover, there was no direct correlation between the IVs and osteoporosis, ensuring the validity of the instrumental variables in this study.

### Causal effects of HBV infection on the development of osteoporosis

**Figure 2:**
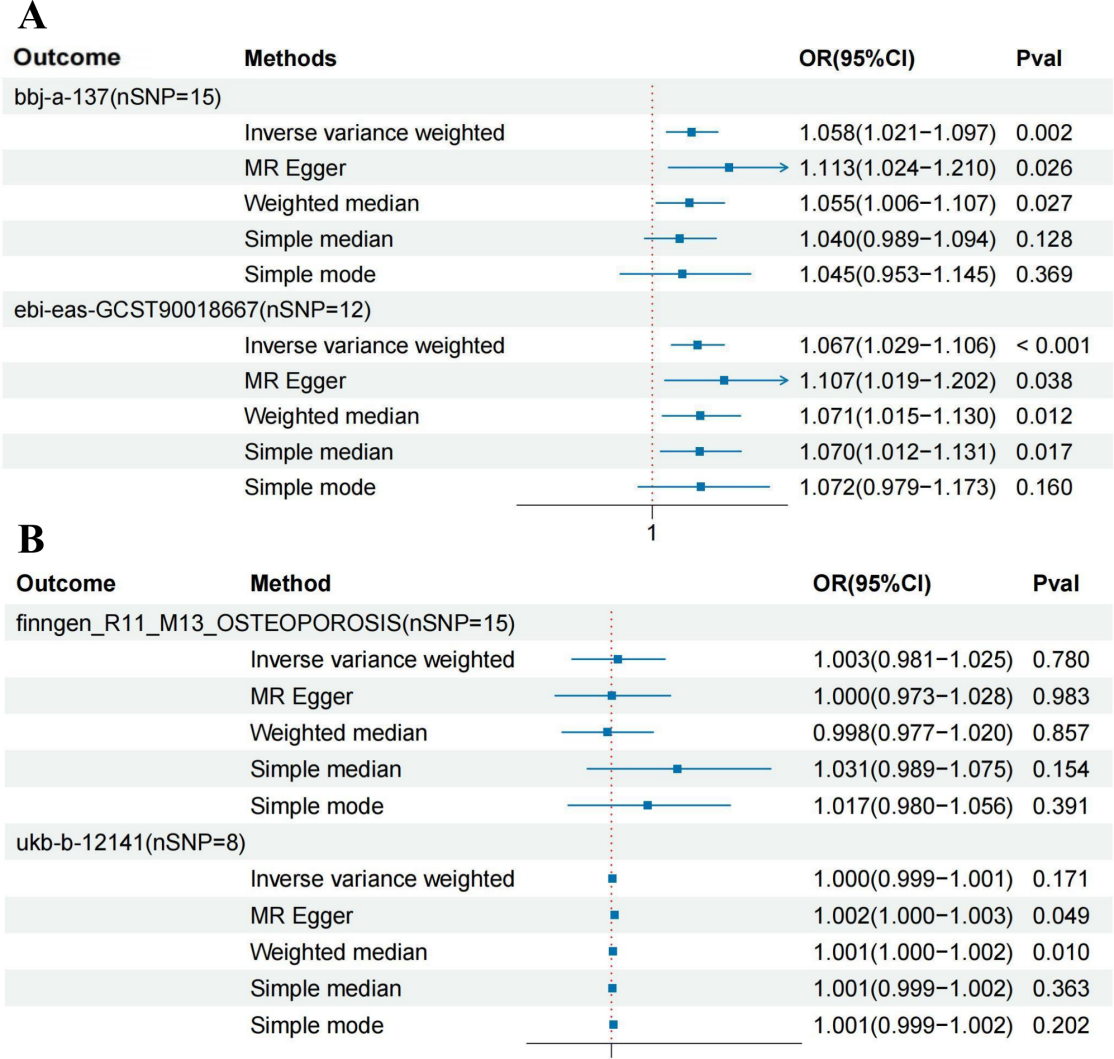
Forest plot showing the causal relationship between hepatitis B virus infection and osteoporosis. **(A)** East Asian population (‘bbj-a-99’ on ‘bbj-a-137’ AND ‘bbj-a-99’ on ‘ebi-eas-GCST90018667’). **(B)** European population (‘ebi-a-GCST006355’ on ‘finngen_R11_M13_OSTEOPOROSIS’ AND ‘ebi-a-GCST006355’ on ‘ukb-b-12141’). HBV, hepatitis B virus; MR, Mendelian randomization; IVW, inverse variance weighted; OR, odds ratio; CI, confidence intervals.

**Figure 3:**
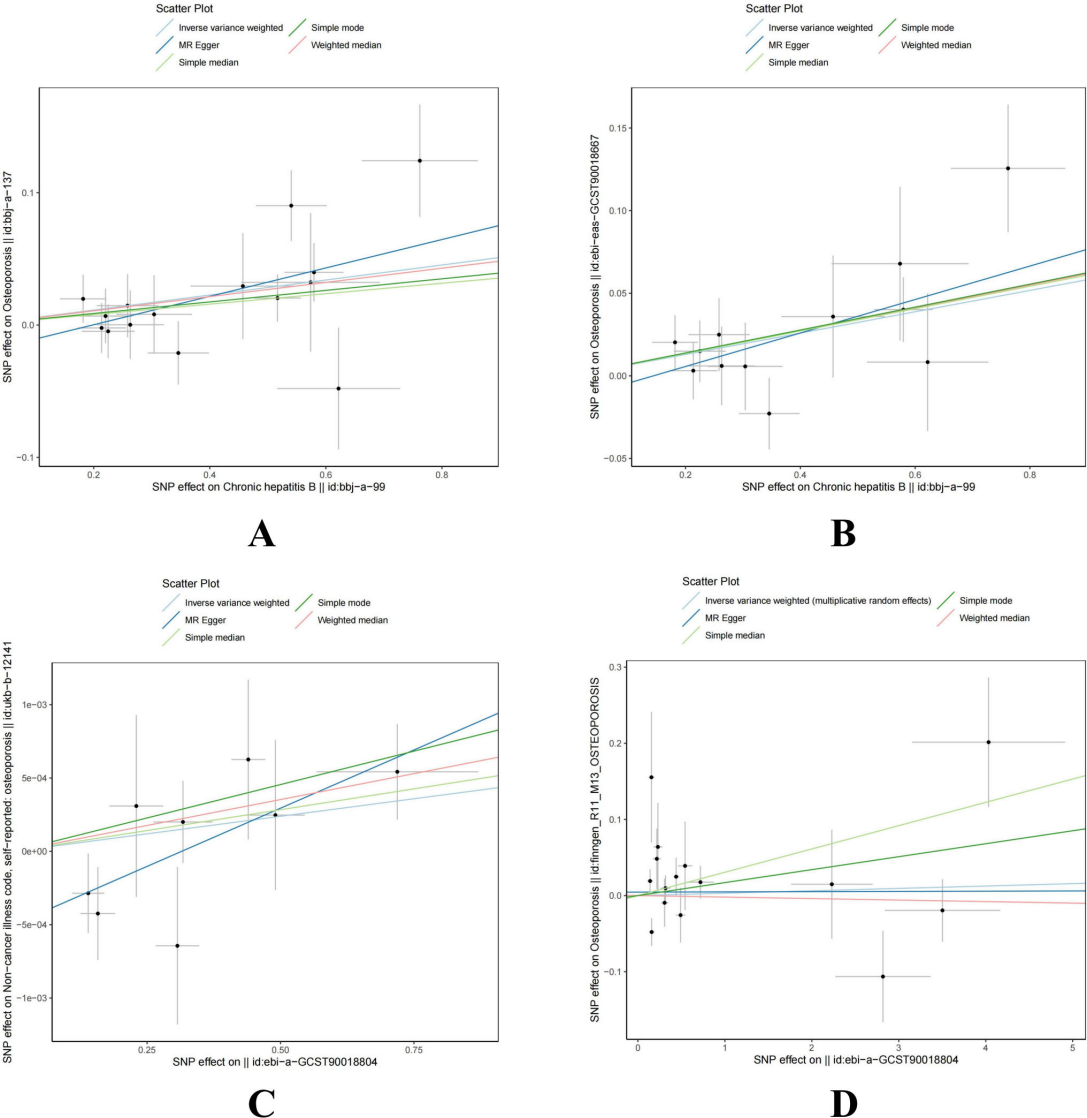
Scatter plot of genetic causality between hepatitis B virus infection and osteoporosis using different MR methods. **(A)** East Asian population (‘bbj-a-99’ on ‘bbj-a-137’). **(B)** East Asian population (‘bbj-a-99’ on ‘ebi-eas-GCST90018667’). **(C)** European population (‘ebi-a-GCST006355’ on ‘ukb-b-12141’). **(D)** European population (‘ebi-a-GCST006355’ on ‘finngen_R11_M13_OSTEOPOROSIS’). MR, Mendelian randomization; IVW, inverse variance weighted; SNP, single nucleotide polymorphism; HBV, hepatitis B virus.

Mendelian randomization analysis revealed a significant causal relationship between HBV infection and osteoporosis in the East Asian population, with consistent results across two different datasets. In the bbj-a-137 dataset, where osteoporosis was the outcome, the results were as follows: IVW (OR = 1.058, 95% CI = 1.021 to 1.097, P = 0.002), MR Egger (OR = 1.113, 95% CI = 1.024 to 1.210, P = 0.026), Weighted median (OR = 1.055, 95% CI = 1.006 to 1.107, P = 0.027), Simple median (OR = 1.040, 95% CI = 0.989 to 1.094, P = 0.128), and Simple mode (OR = 1.045, 95% CI = 0.953 to 1.145, P = 0.369). In the GCST90018667 dataset, where osteoporosis was also the outcome, the results were as follows: IVW (OR = 1.067, 95% CI = 1.029 to 1.106, P < 0.001), MR Egger (OR = 1.107, 95% CI = 1.019 to 1.202, P = 0.038), Weighted median (OR = 1.071, 95% CI = 1.015 to 1.130, P = 0.012), Simple median (OR = 1.070, 95% CI = 1.012 to 1.131, P = 0.017), and Simple mode (OR = 1.072, 95% CI = 0.979 to 1.173, P = 0.160). The main results for the East Asian population are shown in **Figure 2A**. However, this relationship did not show statistical significance in the European population. In the Finnish osteoporosis dataset, the IVW result was (OR = 1.003, 95% CI = 0.981 to 1.025, P = 0.780), and in the UKB osteoporosis dataset, the IVW result was (OR = 1.000, 95% CI = 0.999 to 1.001, P = 0.171). The remaining results are shown in **Figure 2B**. The scatter plots were all presented in **Figure 3**.

### Sensitivity analysis results

The sensitivity analysis of this study included tests for heterogeneity and horizontal pleiotropy (**Table 2**). In the East Asian population, both IVW and MR Egger methods were employed to assess heterogeneity among the IV estimates. No significant heterogeneity was observed, indicating the robustness of the results (bbj-a-137: IVW Q = 17.414, p = 0.235; MR Egger Q = 15.390, p = 0.284; ebi-eas-GCST90018667: IVW Q = 10.690, p = 0.470; MR Egger Q = 9.759, p = 0.462). Additionally, pleiotropy testing for the effect of HBV infection on osteoporosis showed no significant horizontal pleiotropic bias, as indicated by both MR Egger regression intercept analysis and the MR-Presso global test (bbj-a-137: Egger intercept = -0.021, MR Egger p = 0.214, RSS = 20.555, Global test p = 0.251; ebi-eas-GCST90018667: Egger intercept = -0.014, MR Egger p = 0.357, RSS = 3.223, Global test P = 0.458). Funnel plots for both IVW and MR Egger also suggested no horizontal pleiotropy (**Supplementary Figure S2**). Furthermore, a leave-one-out sensitivity analysis revealed that removal of any single SNP did not significantly alter the results, with all lines remaining on the same side of zero (**Supplementary Figure S1**). The same sensitivity analysis was also conducted in the European population; while potential heterogeneity and pleiotropy were observed, these results were not statistically significant, and therefore no further analysis was conducted.

**Table 2:** Sensitivity analysis of the causal association between hepatitis B virus infection and the risk of osteoporosis.

Exposures	Outcomes	Heterogeneity						Pleiotropy				
		IVW			MR Egger			MR Egger			MR-Presso	
		Q	Q_df	Q_pval	Q	Q_df	Q_pval	egger-intercept	se	pval	RSS	Global test Pval
bbj-a-99	bbj-a-137	17.414	14	0.235	15.390	13	0.284	-0.021	0.016	0.214	20.555	0.251
ebi-a-GCST006355	ukb-b-12141	21.436	16	0.162	21.209	15	0.130	0.00017	0.00042	0.694	24.165	0.156
bbj-a-99	GCST90018667	10.690	11	0.470	9.759	10	0.462	-0.014	0.015	0.357	3.223	0.458
ebi-a-GCST006355	finngen_R11_M13_ OSTEOPOROSIS	26.571	14	0.022	26.302	13	0.015	0.005	0.012	0.721	36.612	0.049

IVW, inverse variance weighted; RSS, residual sum of squares.

### Bayesian co-localization analysis

**Figure 4:**
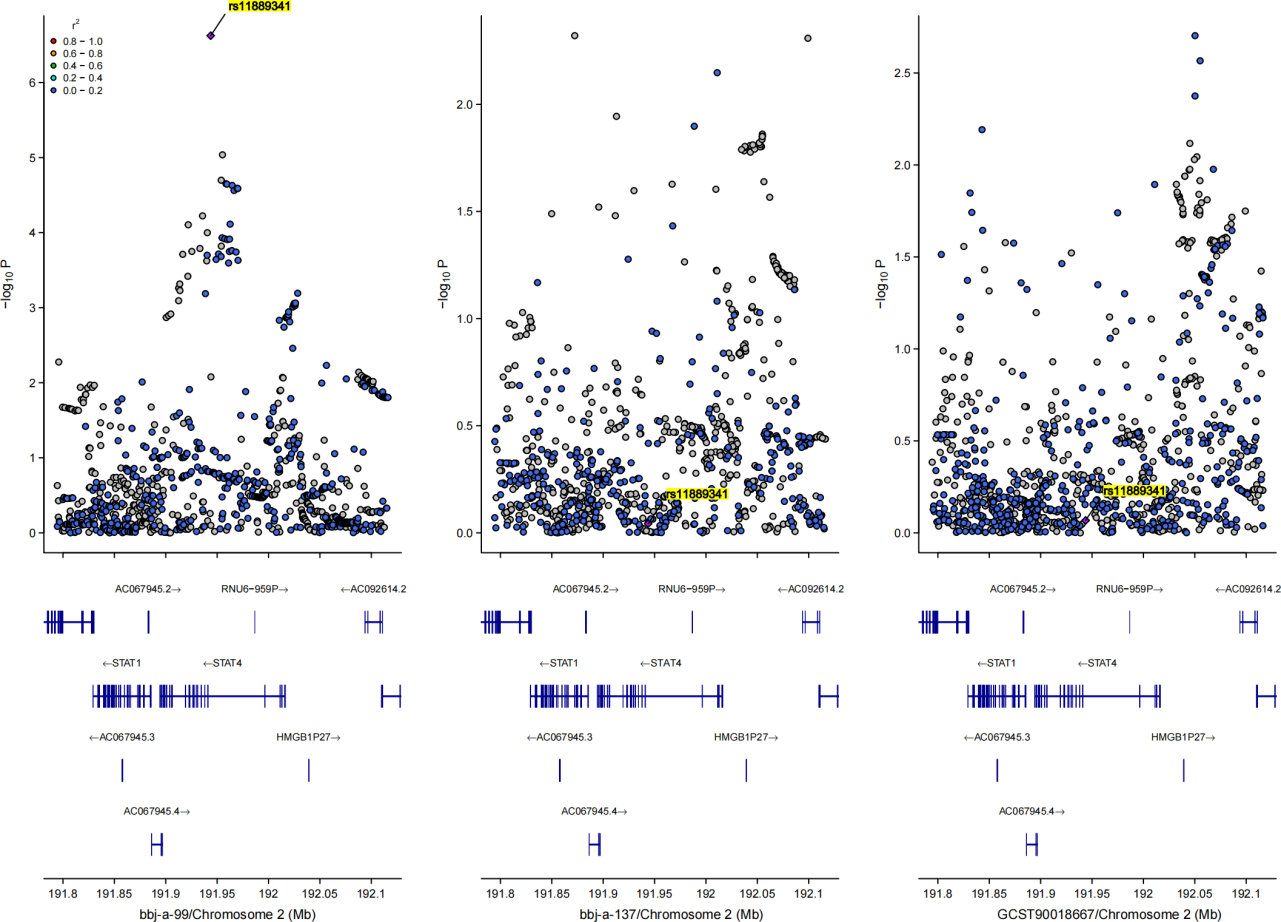
Bayesian co-localization analysis results for the causal relationship between hepatitis B virus and osteoporosis in East Asian population, rs11889341 was highlighted.

Since the results in the East Asian population showed a positive causal relationship between HBV infection and osteoporosis, we conducted a GWAS-GWAS colocalization analysis for both exposure and outcome in this population. **Figure 4** illustrates the distribution of SNPs across the three datasets used in the analysis. The colocalization results indicated that the rs11889341 locus may be a key site for the association between HBV infection and osteoporosis in the East Asian population. Specifically, the colocalization analysis between bbj-a-99 and bbj-a-37 at rs11889341 yielded a PPH4 value of 0.793, with results for other loci presented in **Supplementary Table S3**. Additionally, the colocalization analysis between bbj-a-99 and ebi-eas-GCST90018667 at rs11889341 yielded a PPH4 value of 0.815, with further results shown in **Supplementary Table S4**. Notably, rs11889341 is located in the gene region of the signal transducer and activator of transcription 4 (STAT4) on chromosome 2. Therefore, we speculate that STAT4 may be a key molecule mediating the causal relationship between HBV infection and osteoporosis.

## Discussion

To our knowledge, this is the first MR study to investigate the potential causal relationship between HBV infection and osteoporosis. In the presented study, we conducted MR analyses in East Asian and European populations, respectively, intending to comprehensively explore the causal relationship between HBV infection and osteoporosis. We found that genetically predicted HBV infection was strongly associated with an elevated prevalence of osteoporosis in East Asian individuals. However, no effect of HBV infection on osteoporosis was observed in European populations. In addition, the evidence provided by the sensitivity analysis further supported the reliability and stability of our findings. Interestingly, we searched for the shared genetic variant rs11889341 (located in the gene region of STAT4) between HBV infection and osteoporosis in a given genomic region by performing Bayesian co-localization analysis, confirming the potential of a shared genetic basis between HBV infection and osteoporosis.

STAT4, a key mediator of inflammation and tumor development, is involved in various signaling pathways, and after phosphorylation, it dimerizes and translocates to the nucleus to act as a transcription factor (26). Recent studies have shown that STAT4 expression is positively correlated with HBV, particularly the rs7574865 genotype, which may increase the incidence of chronic HBV infection (27). Notably, Lu et al. (28) found that rs11889341 in STAT4 was associated with HBV infection, aligning with our co-localization results. STAT4 also plays a crucial role in the activation of HBV-specific CTLs, which are essential for controlling HBV infection (29, 30). Additionally, Chen et al. (31) showed that stimulating the JAK2/STAT4 pathway increased CTL activity and inhibited HBV replication. Interestingly, STAT4 has also been linked to osteoporosis. Studies using RT-qPCR, Elisa, and Mendelian randomization demonstrated elevated STAT4 expression in osteoporosis and its positive correlation with IL-2 (32), a known contributor to osteoporosis. Furthermore, single-cell analysis showed a negative correlation between STAT4 and activated dendritic cells (DCs), which differentiate into osteoclasts and promote bone resorption, further linking STAT4 to osteoporosis (33). These findings suggest that STAT4 may enhance osteoporosis risk by facilitating IL-2 production and promoting osteoclast activity. In summary, although not many studies have been conducted on the effect of STAT4 in HBV and osteoporosis, especially the relationship between STAT4 and osteoporosis. However, the current study suggested that STAT4 may be a novel biomarker for the prevention and treatment of HBV and osteoporosis, which is in agreement with our findings. Importantly, our results combined with previous studies hinted that STAT4 may be a pivotal molecule in the process of increasing the prevalence of osteoporosis under HBV infection (**Supplementary Figure S3**). However, this is still our speculation and additional researches are required to ascertain the molecular mechanism by which STAT4 regulated the process that HBV infection contributed to the development of osteoporosis.

Another interesting finding from our study was that the causal association between HBV infection and osteoporosis was observed exclusively in the Asian population. One reason for this is that the prevalence of HBV infection in the Asian population is likely much higher than in the European population, and the epidemiological burden in Asia results in a larger number of individuals who are positive for the virus, which leads to a higher overall proportion of individuals with osteoporosis (34). In this context, the pressure for antiviral treatment in the Asian population has also increased significantly, and antiviral therapy may have detrimental effects on bone metabolism, contributing to decreased bone mineral density (35). Additionally, differences in lifestyle and diet may also contribute to the increased susceptibility to osteoporosis following HBV infection. For example, diet and lifestyle habits can affect vitamin D levels, which in turn influence bone mineral density. While this study primarily focused on genetic variations, it is important to note that lifestyle and dietary factors are environmental influences. Compared to Europeans, Asians generally have lower levels of vitamin D, which may unintentionally increase the risk of osteoporosis in HBV-infected individuals (36).

Our study has several distinct strengths. We utilized the most up-to-date and comprehensive GWAS data available on HBV infection and osteoporosis, and explored the potential causal relationship between HBV infection and osteoporosis in two populations, East Asia and Europe, respectively. Moreover, corresponding homogeneous populations were used in both population studies, which minimized potential population heterogeneity and increased the applicability of the results. In addition, our study focused on whether common mechanisms underlie the causal relationship between HBV infection and osteoporosis, which will assist in the design of targeted measures to combat HBV infection and osteoporosis. However, there are limitations to this study. First, relying on existing data resources, we only used data from GWAS to investigate the causal relationship between HBV infection and osteoporosis, this data source greatly limits the scope of our analysis, restricting it to the exploration of the broad causal relationship between the two diseases. Given that our data heavily relies on raw GWAS data, we were unable to conduct further subgroup analyses, such as by age, gender, and other factors. These variables are crucial for understanding osteoporosis, and therefore, further investigations into these relationships should be explored using more specific clinical data. Future studies could consider supplying more favorable evidence by using other data sources for validating and establishing a causal relationship. Besides, although we conducted MR analyses in two populations, East Asia and Europe, these results may not be extended to the whole population due to regional and ethnic differences.

## Conclusion

We established a causal relationship that HBV infection may increase the risk of osteoporosis development and searched for common genetic variants between HBV infection and osteoporosis with this MR study. Our findings may have significant implications for the prevention and clinical management of these diseases.

## Data Availability

The original contributions presented in the study are included in the article/**Supplementary Material**. Further inquiries can be directed to the corresponding authors.

## Supplementary material

Supplementary Figure 1 “Leave-one-out” plots for the causal association between hepatitis B virus infection and osteoporosis. **(A)** East Asian population (‘bbj-a-99’ on ‘bbj-a-137’). **(B)** East Asian population (‘bbj-a-99’ on ‘ebi-eas-GCST90018667’). **(C)** European population (‘ebi-a-GCST006355’ on ‘ukb-b-12141’). **(D)** European population (‘ebi-a-GCST006355’ on ‘finngen_R11_M13_OSTEOPOROSIS’).

Supplementary Figure 2 Funnel plots for the causal association between hepatitis B virus infection and osteoporosis. **(A)** East Asian population (‘bbj-a-99’ on ‘bbj-a-137’). **(B)** East Asian population (‘bbj-a-99’ on ‘ebi-eas-GCST90018667’). **(C)** European population (‘ebi-a-GCST006355’ on ‘ukb-b-12141’). **(D)** European population (‘ebi-a-GCST006355’ on ‘finngen_R11_M13_OSTEOPOROSIS’).

Supplementary Figure 3 Molecular mechanism of STAT4 as a key molecule in HBV infection and osteoporosis. Fusion proteins Ub-HBcAg-CTP and CTP-HBcAg-Tapasin could up-regulate the expression levels of JAK2, Tyk2, STAT1, and STAT4 in T-lymphocytes, which in turn activated the JAK2/STAT4 signaling pathway. The activation of the JAK2/STAT4 signaling pathway enhanced the percentage of CTLs, inducing HBV-specific CTLs immune response and ultimately combating HBV infection. In addition, STAT4 may add to the risk of osteoporosis through two pathways: inducing DCs to be derived into osteoclasts and activated, promoting IL-2 production. Notably, Palbociclib may suppress osteoporosis by mediating STAT4 mRNA degradation through its action on miR-141-3p. Furthermore, CCL12 could stimulate RANKL production in bone marrow stromal cells of acute lung injury mice via the CCR 2/JAK 2/STAT 4 axis, which consequently promoted trabecular bone loss and elevated bone resorption *in vivo*, and manifested as osteoporosis. HBV, hepatitis B virus; Ub, ubiquitin; HBcAg, hepatitis B core antigen; CTP, cytoplasmic transduction peptide; JAK2, janus kinase 2; STAT4, signal transducer and activator of transcription 4; CCL12, chemokine (C-C motif) ligand 12; CCR2, chemokine (C-C motif) receptor 2; RANKL, receptor activator of nuclear factor kappa-B ligand; RANK, receptor activator of nuclear factor kappa-B; CTLs, cytotoxic T-lymphocytes; IL-2, interleukin-2.

Supplementary Table 1 All SNP data used for the analysis of the Asian population.

Supplementary Table 2 All SNP data used for the analysis of the European population.

Supplementary Table 3 Results of the GWAS-GWAS colocalization analysis for the Asian population (‘bbj-a-99’ on ‘bbj-a-137’).

Supplementary Table 4 Results of the GWAS-GWAS colocalization analysis for the Asian population (‘bbj-a-99’ on ‘ebi-eas-GCST90018667’).

Supplementary Table 5 STROBE-MR-checklist.
