# Supplementary figures and images for: Causal associations between osteoporosis and HBV infection across Asian and European populations: evidence from Mendelian randomization and colocalization analysis
Source: Front Endocrinol (Lausanne). 2025 Jan 22;15:1419303. doi: 10.3389/fendo.2024.1419303 (PMC11794126; doi:10.3389/fendo.2024.1419303)

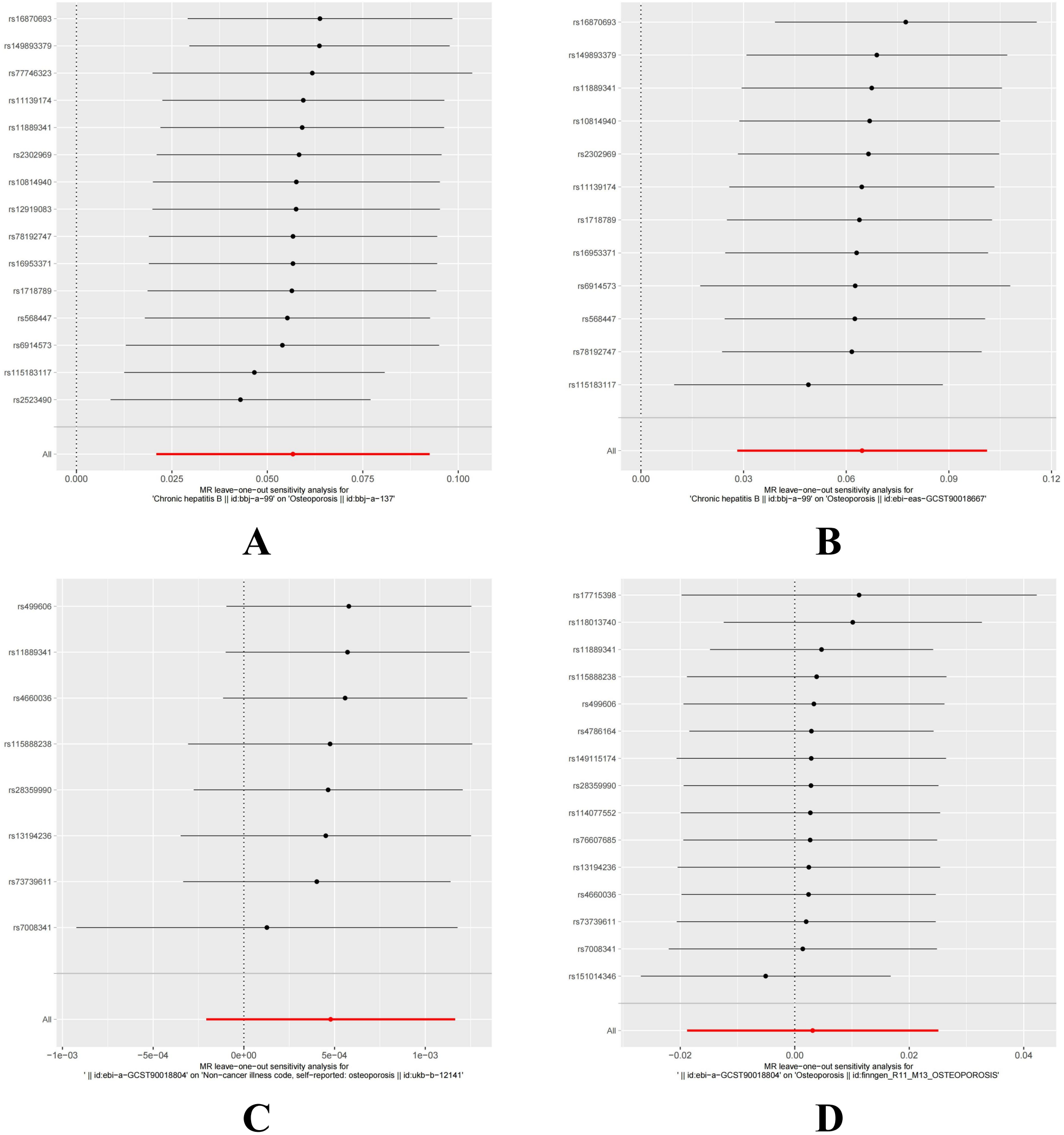

Supplement: Supplementary Figure 1 — “Leave-one-out” plots for the causal association between hepatitis B virus infection and osteoporosis. (A) East Asian population (‘bbj-a-99’ on ‘bbj-a-137’). (B) East Asian population (‘bbj-a-99’ on ‘ebi-eas-GCST90018667’). (C) European population (‘ebi-a-GCST006355’ on ‘ukb-b-12141’). (D) European population (‘ebi-a-GCST006355’ on ‘finngen_R11_M13_OSTEOPOROSIS’). [file Image1.tif]

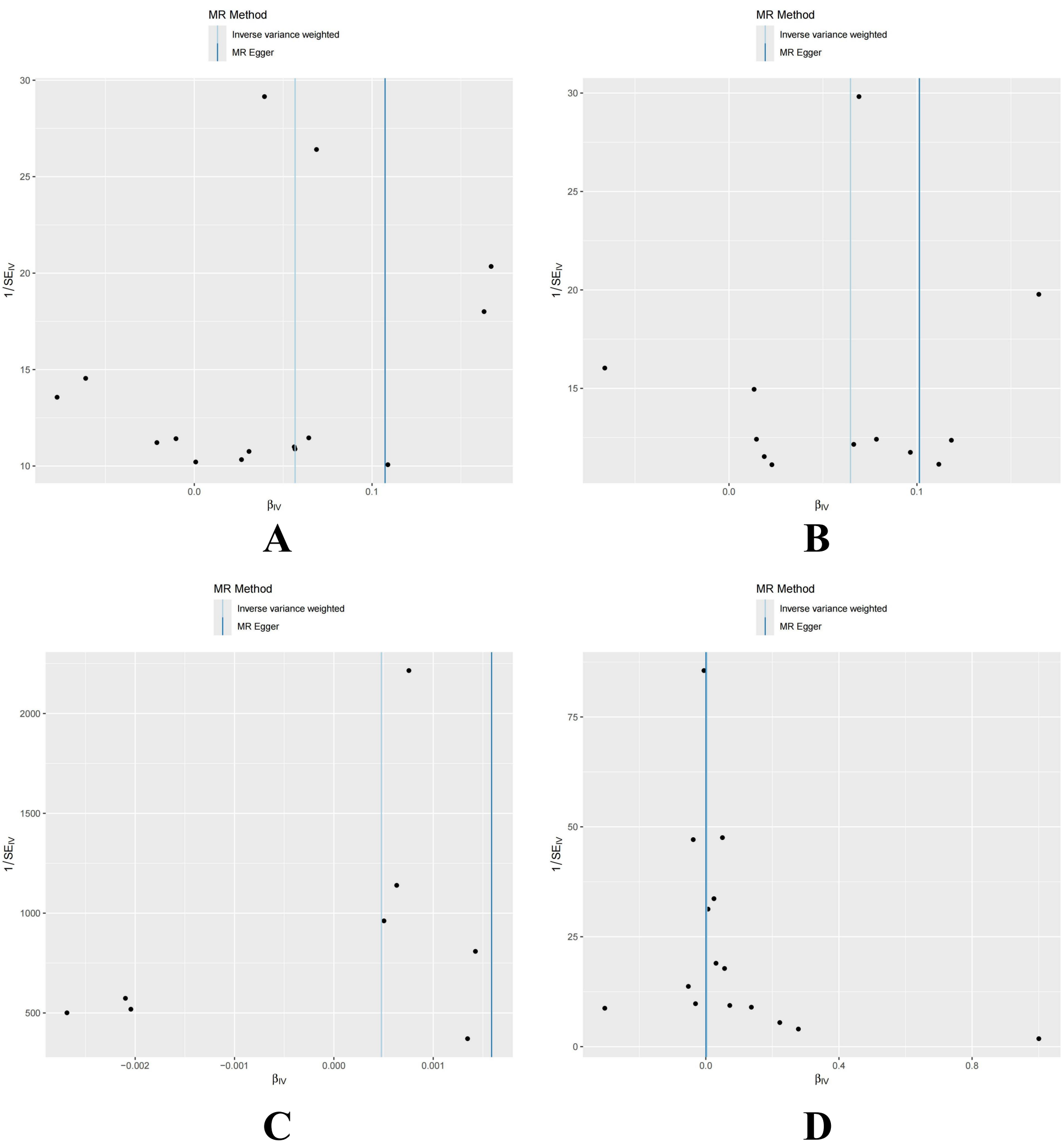

Supplement: Supplementary Figure 2 — Funnel plots for the causal association between hepatitis B virus infection and osteoporosis. (A) East Asian population (‘bbj-a-99’ on ‘bbj-a-137’). (B) East Asian population (‘bbj-a-99’ on ‘ebi-eas-GCST90018667’). (C) European population (‘ebi-a-GCST006355’ on ‘ukb-b-12141’). (D) European population (‘ebi-a-GCST006355’ on ‘finngen_R11_M13_OSTEOPOROSIS’). [file Image2.tif]

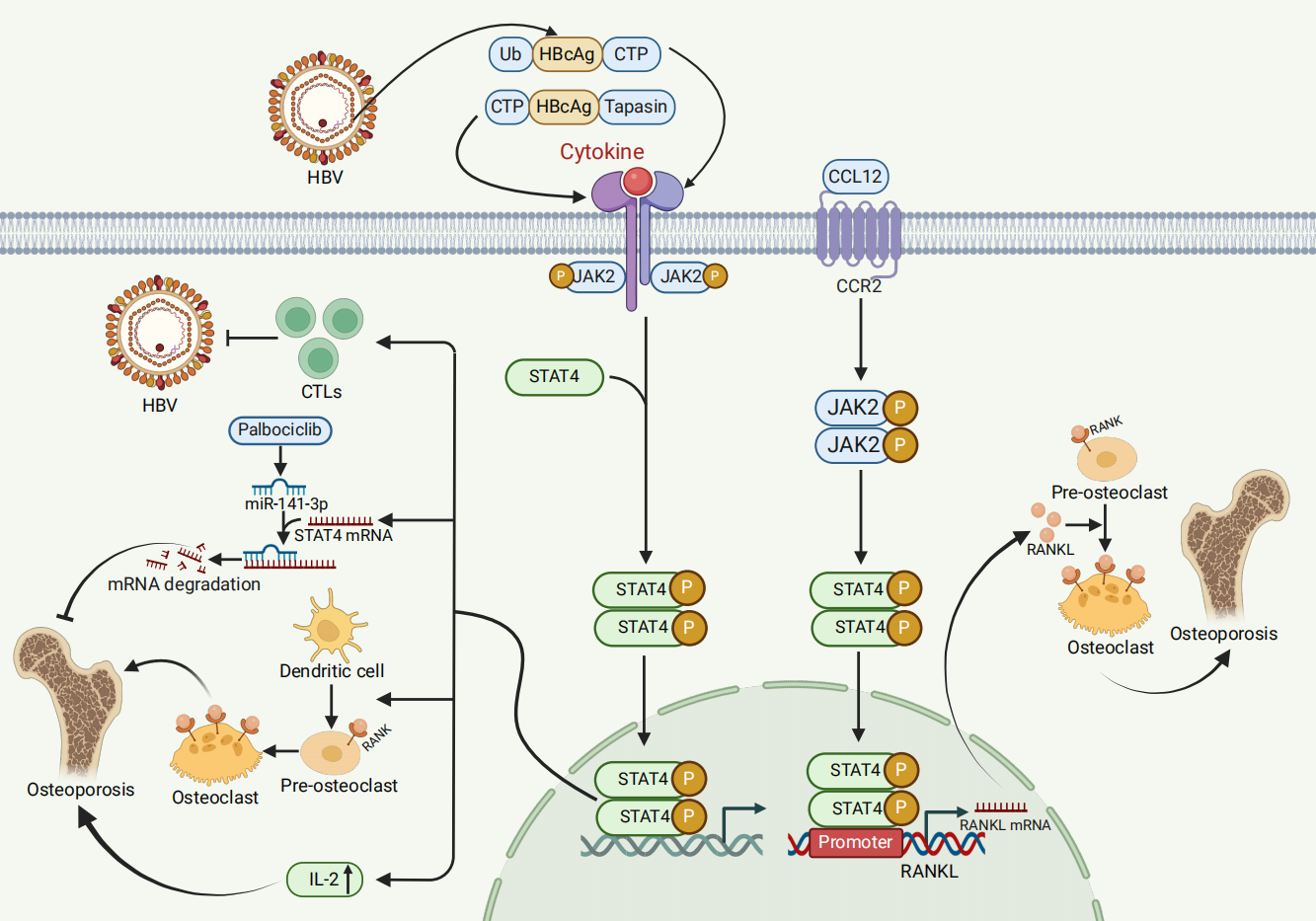

Supplement: Supplementary Figure 3 — Molecular mechanism of STAT4 as a key molecule in HBV infection and osteoporosis. Fusion proteins Ub-HBcAg-CTP and CTP-HBcAg-Tapasin could up-regulate the expression levels of JAK2, Tyk2, STAT1, and STAT4 in T-lymphocytes, which in turn activated the JAK2/STAT4 signaling pathway. The activation of the JAK2/STAT4 signaling pathway enhanced the percentage of CTLs, inducing HBV-specific CTLs immune response and ultimately combating HBV infection. In addition, STAT4 may add to the risk of osteoporosis through two pathways: inducing DCs to be derived into osteoclasts and activated, promoting IL-2 production. Notably, Palbociclib may suppress osteoporosis by mediating STAT4 mRNA degradation through its action on miR-141-3p. Furthermore, CCL12 could stimulate RANKL production in bone marrow stromal cells of acute lung injury mice via the CCR 2/JAK 2/STAT 4 axis, which consequently promoted trabecular bone loss and elevated bone resorption in vivo, and manifested as osteoporosis. HBV, hepatitis B virus; Ub, ubiquitin; HBcAg, hepatitis B core antigen; CTP, cytoplasmic transduction peptide; JAK2, janus kinase 2; STAT4, signal transducer and activator of transcription 4; CCL12, chemokine (C-C motif) ligand 12; CCR2, chemokine (C-C motif) receptor 2; RANKL, receptor activator of nuclear factor kappa-B ligand; RANK, receptor activator of nuclear factor kappa-B; CTLs, cytotoxic T-lymphocytes; IL-2, interleukin-2. [file Image3.tif]
